# Barriers to women's participation in inter-conceptional care: a cross-sectional analysis

**DOI:** 10.1186/1471-2458-12-93

**Published:** 2012-02-01

**Authors:** Vijaya K Hogan, M Ahinee Amamoo, Althea D Anderson, David Webb, Leny Mathews, Diane Rowley, Jennifer F Culhane

**Affiliations:** 1 Gillings School of Global Public Health, University of North Carolina at Chapel Hill, Chapel Hill, NC, USA; 2 Children's Hospital of Philadelphia, Philadelphia, USA; 3 Mailman School of Public Health, Columbia University, New York, NY, USA; 4 Department of Maternal and Child Health, Gillings School of Global Public Health, University of North Carolina at Chapel Hill, CB# 7445, Room 425 Rosenau, 421 Pittsboro Street, Chapel Hill, NC 27599, USA

**Keywords:** Prematurity, Preterm birth, African American women, Pregnancy, Perinatal periods of risk, Health care participation, Infant mortality, Preventive care, Access to care, Utilization of care, Preconception care

## Abstract

**Background:**

We describe participation rates in a special interconceptional care program that addressed all commonly known barriers to care, and identify predictors of the observed levels of participation in this preventive care service.

**Methods:**

A secondary analysis of data from women in the intervention arm of an interconceptional care clinical trial in Philadelphia (n = 442). Gelberg-Andersen Behavioral Model for Vulnerable Populations to Health Services (herein called Andersen model) was used as a theoretical base. We used a multinomial logit model to analyze the factors influencing women's level of participation in this enhanced interconceptional care program.

**Results:**

Although common barriers were addressed, there was variable participation in the interconceptional interventions. The Andersen model did not explain the variation in interconceptional care participation (Wald ch sq = 49, *p = *0.45). *Enabling *factors *(p *= 0.058), *older maternal age (p = 0.03) *and *smoking (p = < 0.0001) *were independently associated with participation.

**Conclusions:**

Actively removing common barriers to care does not guarantee the long-term and consistent participation of vulnerable women in preventive care. There are unknown factors beyond known barriers that affect participation in interconceptional care. New paradigms are needed to identify the additional factors that serve as barriers to participation in preventive care for vulnerable women.

## Background

### Problem statement

Preterm birth remains a leading cause of infant mortality, particularly for African American women. In fact, the impact of premature births on infant mortality may be larger than indicated by standard preterm birth rates. A "preterm-related infant mortality rate" is a measure of aggregate deaths across all underlying causes documented by ICD-9. At least 75% of the preterm related deaths occur among infants born less than 37 weeks gestation [1,2]. The preterm related infant mortality rate for blacks (6.01) is 3.4 times higher than for whites (1.79), and in fact, the black preterm related infant mortality rate is higher than the overall white IMR [3].

The various underlying causes of infant mortality pose a challenge in finding appropriate intervention approaches. The current approach relies heavily on the provision of prenatal care, which has not been effective in reducing the preterm-related causes of IM, nor in reducing the disparity between Blacks and Whites.

**Figure 1:**
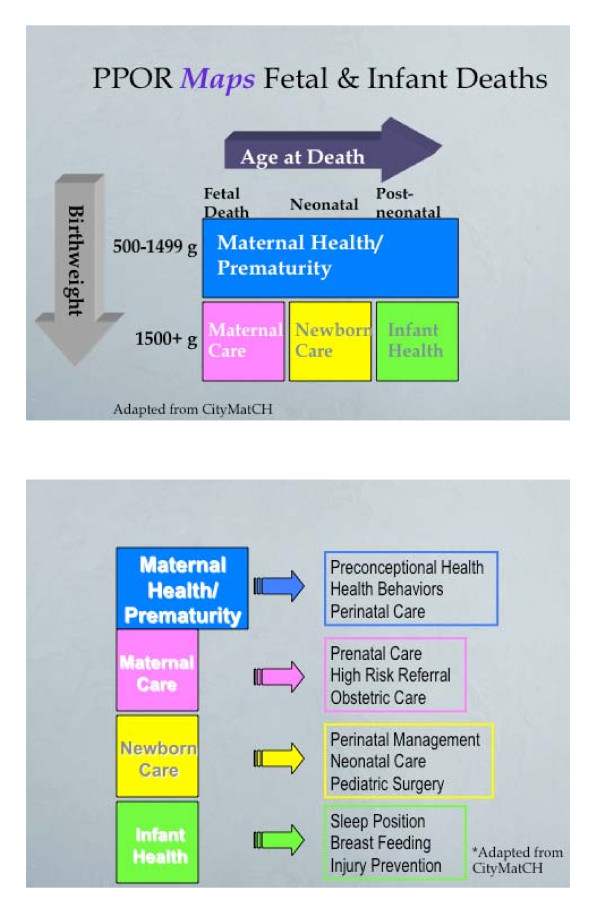
**Mapping perinatal periods of risk to opportunities for prevention.** A: shows the birthweight and age at death distribution that segments periods of risk. B: maps these periods of risk to existing intervention opportunities

In the late 1990s, several state and local health departments adopted a new analysis approach to identify priority areas of action in reducing infant mortality and for addressing racial disparity in preterm birth [4]. This method, called Perinatal Periods of Risk (PPOR), classifies all infant deaths to a specific "period of risk" where the opportunity for prevention is greatest. The periods of risk are determined based on age at death and birth weight. There are 4 strata representing opportunities for prevention: (1) maternal health/preconceptional care (MH/P), (2) prenatal care, (3) newborn care, and (4) post partum child health care (Figure 1). Six peer-reviewed studies using the PPOR method to examine feto-infant mortality (FIM) and racial/ethnic variations in FIM throughout the U.S. have been published [5-9]. The overall FIM rate ranged from 9.0 to 12.7 feto-infant deaths (per 1000 live births plus fetal deaths) for studies that reported this indicator [5-9]. Using various reference groups, the proportion of excess feto-infant deaths were in the MH/P category and ranged from 33% to over 50% of infant deaths [7-10].

In addition to mapping FIM, four of the six studies also compared FIM rates and the contribution of excess death to the four categories of mortality risk across racial and ethnic subpopulations. A study conducted in North Carolina, reported 14.7 feto-infant deaths per 1,000 (live births and fetal deaths) for African Americans compared to 6.0 for whites (excess risk of death is 8.7 per 1,000 live births) [8]. The PPOR analysis also shows that the "Maternal health/prematurity" category (Figure 1b) contributed the largest share of the total African American FIM rate compared to other racial and ethnic groups [6,8-10]. Two of the studies indicate that nearly half of the excess feto-infant deaths among African Americans could be ascribed to the MH/P category, indicating that the best opportunity for prevention of over half the excess infant deaths among Blacks was during the preconception period [8,9]. The high excess risk of deaths in the MHP category has been consistent for African American populations across the US. Consistent with other findings, the racial disparity (between Blacks and Whites) in infant mortality is attributed to the disparity in premature rates.

There is growing consensus that care needs to be delivered to women starting in the period *before *pregnancy in order to decrease the excess risks of prematurity for African American women [11]. Care delivered during the period before pregnancy is called *preconceptional (PCC) or interconceptional care *(ICC). "Preconceptional" refers to women who are in their first pregnancy, and "interconceptional" refers to women who have had previous pregnancies. ICC addresses pregnancy risks before the disease pathways have advanced too far to be reversed. It allows a larger window of time for risk reduction to occur and thus increases the number of healthier women entering pregnancy. Clinical guidelines recommending specific services to be included in ICC have been published [12]. These services focus on evidence-based preventive care as well as screening and treatment for existing risk conditions. However, barriers that prevent women from full participation in care must be taken into consideration. The factors that influence the participation of women in organized *prenatal care *have been thoroughly studied. Factors such as insurance status, transportation, and childcare have been shown to influence access to prenatal care [13]. What is unknown is--if these factors are addressed, will it facilitate full participation and reduce racial/ethnic inequalities in access to interconceptional care? Factors that influence participation in interconceptional care have not previously been studied. This study examines the predictors of participation in interconceptional care.

## Methods

### Theoretic framework

This analysis uses the Andersen's Behavioral Model of Utilization of Care to predict factors influencing women's access to ICC [14]. This model is well documented and widely used to determine predictors of access to care. The framework posits that access to and utilization of care can be predicted by a predisposition of people to use services, factors that enable or impede this use, a person's perception of need for care, and systems factors. Some of the specific 'predisposing' factors include age, gender, education, and ethnicity. 'Enabling' factors include having insurance; 'Need' factors include perceptions about health; and 'structural/systems' factors include such things as transportation and childcare. Recent developments in the tool have taken into account additional factors affecting vulnerable populations, including immigration status, acculturation, neighborhood conditions, psychological resources, housing mobility, mental illness, competing needs and food sources.

### Study population

We conducted a secondary analysis of subjects participating in a randomized clinical/behavioral trial of an interconceptional preterm birth risk reduction intervention in Philadelphia [15]. Resident women experiencing a preterm birth at < 34 weeks of gestation were enrolled in the parent study. Women in the *intervention arm *received a series of intensive interventions designed to reduce their risks related to inflammatory pathways leading to a subsequent preterm birth. Six specific risks were addressed because of their common contribution to the inflammatory pathways to premature birth. These include genito-urinary infection, weight control, depression, housing inadequacy (stressor), smoking cessation and periodontal disease. Interventions on risks contributing to an inflammatory pathway (smoking, depression, infectious disease burden and maternal stress, and achieving an appropriate BMI), were introduced to decrease systemic inflammation and risk of repeat PTB.

### Parent study data collection

At the hospital visit, all participants were interviewed after delivery and prior to discharge to elicit demographic and other information. Once the maternal interview was completed, participants were randomly assigned to either intervention or usual care. For women assigned to the intervention group, the first study visit was scheduled within four weeks of discharge from the hospital. The one-month post partum visit for intervention group women was conducted at Drexel University. For women with multiple risk factors, interventions were delivered in stages over the 2- year intervention period. All intervention services scheduled and received were carefully documented and entered into tracking software. In addition, the services were delivered in a method that removed as many barriers as possible given existing knowledge of known barriers to care. All services were free of charge; women were provided with transportation, childcare and social support as needed; and appointment reminders were provided. In some cases, financial incentives were provided.

All women enrolled in the intervention arm of the parent study were included in this analysis. (n = 442). This study was approved by the Drexel University and the UNC-CH IRB.

The goals of this analysis were to identify and validate specific factors that adversely impact on women's ability to participate in interconceptionally delivered preterm birth prevention interventions, once all known barriers to care are addressed.

We used the modified Andersen Behavioral model to determine key predictors of access to interconceptional care for women at high risk of PTB. Specifically, we wanted to assess (1) *how components of the Andersen Behavioral Model differed in this inner city urban population by level of participation after the known barriers were removed, and (2) to what extent the components of the Andersen Behavioral Model predict utilization of interconceptional care interventions for women at high risk of a subsequent preterm birth*.

### Independent variables

The independent predictors include **P**redisposing factors, **E**nabling factors, **N**eed factors and **S**ystems factors. These will herein be collectively referred to as *PENS*.

Predisposing variables are operationalized using age (continuous), marital status (married/not married), education (< HS, HS grad, > HS), family size (1 or 2 members vs 2 or more) and substance use (Y/N for alcohol or drugs).

Enabling Factors are operationalized using insurance status (Y/N), income (categorical), availability of social support (Y/N), neighborhood safety and quality (safe/not safe), and perception of competing needs (Y/N).

Need Factors are operationalized by including perceived health (good/poor), and reported diagnosis of major health problems (Y/N).

System factors are measured as self- report of prior experience with providers (Good/Poor). Some Systems factors defined by the Andersen model are not included in this analysis because they are addressed by the parent study intervention (e.g. transportation, childcare), or because we did not have the data to assess them (homelessness length, language barriers).

### Dependent variable

Utilization of interconceptional care is measured as the number of visits completed divided by the number of visits scheduled between date of enrollment into the parent study and December 30, 2007. Only visits that required the woman to travel to a clinical setting were counted in the denominator. All home visits and phone interventions were excluded, thus this measure does not represent the overall or intervention-specific participation rates of the parent clinical trial. The utilization patterns for in-clinic visits were aggregated for the following 6 parent study interventions: Weight control, infection (vaginal), periodontal disease, housing, smoking cessation and depression. The aggregate counts were divided by the total aggregate number of scheduled visits and the resultant participation rates were grouped into 4 categories for analysis: "None" (did not attend any of the scheduled visits), "Some" (attended 1% to < 50% visits); "Most" (attended 50%-99%); or "All" (attended all (100%) of scheduled visits).

### Statistical analysis

Chi-square analyses were conducted to assess bivariate associations between interconceptional care participation levels and the predictive factors of the Andersen Model. Proportional odds models (POM) were proposed to assess the Andersen Model's predictability, however the proportionality assumptions were not met. Therefore, participation was analyzed as a nominal variable, and a generalized logit model for nominal outcomes was fit [16].

We assessed (a) the collective predictive power of the PENS factors in the utilization of each specific interconceptional care intervention, (b) which *construct *(predisposing, enabling, need or system) has the strongest significant effect, and (c) which specific individual factors within the sets are most predictive of utilization of care. Backward elimination was used to determine significant predictors, with a selection to stay criteria of 0.20. "Attended all visits" was used as the reference group for all analyses. All analyses were conducted using SAS 9.2 [17].

## Results

### Population description

All of the women in the study population had previously experienced a premature birth. Subjects were most likely to be African American (84.7%), have a mean age of 25 years, be unmarried (88.4%), live in households with more than 3 people (69.3%), and have at least a high school education (66.6%). Most women did not engage in high-risk behaviors, such as smoking, drug use or alcohol use (69.7%, 84.7, and 88.1%, respectively). (Table 1) Smoking was the only factor that showed a significant difference by level of participation (*p *= 0.0396, data not shown).

**Table 1:** Demographic description of study population by care utilization (%)

Characteristics	Overall (N = 442)	No visits (N = 78)	0 to 50% (N = 116)	50 to 99% (N = 157)	All visits (N = 91)	Chi-square *P*-value
**Predisposing Factors**						

Race- White	26.0(6.0)	5(6.8)	5(4.4)	12(7.7)	4(4.5)	0.6636
	
Black	366.0(84.7)	62(83.8)	99(86.8)	127(81.9)	78(87.6)	
	
Hispanic	33.0(7.6)	6(8.1)	9(7.9)	14(9.0)	4(4.5)	
	
Other	7.0(1.6)	1(1.4)	1(0.9)	2(1.3)	3(3.4)	
	
Missing Race	10	4	2	2	2	
	
Mean Age in Years (Std)	25(6)	25 (6)	26 (6)	25 (6)	24 (5)	

Marital Status- Not Married	389.0(88.4)	66(84.6)	106(92.2)	139(88.5)	78(86.7)	0.9976
	
Married	51.0(11.6)	12(15.4)	9(7.8)	18(11.5)	12(13.3)	
	
Missing Married	2	0	1	0	1	

Education: < High School	147.0(33.3)	28(35.9)	44(37.9)	50(31.8)	25(27.5)	0.2354
	
High School Education,%	184.0(41.6)	32(41.0)	47(40.5)	67(42.7)	38(41.8)	
	
> = High School Education,%	111.0(25.1)	18(23.1)	25(21.6)	40(25.5)	28(30.8)	

Family Size- 1 or 2 people	134.0(30.7)	21(26.9)	36(31.0)	48(31.6)	29(31.9)	0.5039
	
Family Size- 3 or more people	303.0(69.3)	57(73.1)	80(69.0)	104(68.4)	62(68.1)	
	
Missing Family Size	5	0	0	5	0	

Substance Abuse- No Drug Use	305.0(84.7)	57(87.7)	71(77.2)	110(85.3)	67(90.5)	0.2832
	
Drug Use	55.0(15.3)	8(12.3)	21(22.8)	19(14.7)	7(9.5)	
	
Drug use not collected	82	13	24	28	17	

No Alcohol Use	317.0(88.1)	59(90.8)	79(85.9)	115(89.1)	64(86.5)	0.6661
	
Alcohol Use	43.0(11.9)	6(9.2)	13(14.1)	14(10.9)	10(13.5)	
	
Alcohol info not collected	82	13	24	28	17	

Non-Smoker	301.0(69.7)	57(76.0)	57(51.8)	114(73.1)	73(80.2)	0.0396
	
Smoker	131.0(30.3)	18(24.0)	53(48.2)	42(26.9)	18(19.8)	
	
Missing Smoking	10	3	6	1	0	
	
**Enabling Factors**						

Health Insurance(not Medicaid)	103.0(23.3)	19(24.4)	26(22.4)	31(19.7)	27(29.7)	0.1564
	
Medicaid	323.0(73.1)	54(69.2)	85(73.3)	121(77.1)	63(69.2)	
	
Uninsured	16.0(3.6)	5(6.4)	5(4.3)	5(3.2)	1(1.1)	

Income: Less than $5,000	35.0(7.9)	3(3.8)	12(10.3)	15(9.6)	5(5.5)	0.9569
	
$5,001 to $10,000	75.0(17.0)	9(11.5)	20(17.2)	32(20.4)	14(15.4)	
	
$10,001 to $15,000	49.0(11.1)	10(12.8)	12(10.3)	18(11.5)	9(9.9)	
	
$15,001 to $20,000	42.0(9.5)	7(9.0)	9(7.8)	17(10.8)	9(9.9)	
	
$20,001 to $25,000	55.0(12.4)	12(15.4)	13(11.2)	18(11.5)	12(13.2)	
	
$25,001 to $30,000	28.0(6.3)	5(6.4)	10(8.6)	7(4.5)	6(6.6)	
	
$30,001 to $40,000	25.0(5.7)	3(3.8)	7(6.0)	12(7.6)	3(3.3)	
	
$40,001 to $60,000	35.0(7.9)	9(11.5)	10(8.6)	8(5.1)	8(8.8)	
	
More than $60,000	15.0(3.4)	5(6.4)	4(3.4)	2(1.3)	4(4.4)	
	
Don't Know	81.0(18.3)	15(19.2)	18(15.5)	27(17.2)	21(23.1)	
	
Refused	2.0(0.5)	0(0.0)	1(0.9)	1(0.6)	0(0.0)	

Income(Modeling version)						0.2123
	
> $20,000	201.0(56.0)	29(46.0)	53(54.6)	82(63.6)	37(52.9)	
	
< $20,000	158.0(44.0)	34(54.0)	44(45.4)	47(36.4)	33(47.1)	

No Social Support	116.0(27.3)	19(25.3)	34(30.4)	38(25.0)	25(29.1)	0.9102
	
Social Support	309.0(72.7)	56(74.7)	78(69.6)	114(75.0)	61(70.9)	
	
Missing Social Support	17	3	4	5	5	
	
Neighborhood Safe	209.0(49.3)	41(54.7)	53(47.3)	68(45.0)	47(54.7)	0.9039
	
Neighborhood not safe,	215.0(50.7)	34(45.3)	59(52.7)	83(55.0)	39(45.3)	
	
Missing Neighborhood Safety	18	3	4	6	5	

Perceived competing need	226.0(51.1)	40(51.3)	58(50.0)	79(50.3)	49(53.8)	0.7368
	
No competing Need	216.0(48.9)	38(48.7)	58(50.0)	78(49.7)	42(46.2)	
	
**Need Factors**						

Poor Mental Health	95.0(21.5)	17(21.8)	30(25.9)	29(18.5)	19(20.9)	0.5346
	
Good Mental Health	347.0(78.5)	61(78.2)	86(74.1)	128(81.5)	72(79.1)	

Poor Physical Health	55.0(12.4)	8(10.3)	18(15.5)	20(12.7)	9(9.9)	0.7223
	
Good Physical Health	387.0(87.6)	70(89.7)	98(84.5)	137(87.3)	82(90.1)	

No Major Health Problems	300.0(67.9)	58(74.4)	77(66.4)	105(66.9)	60(65.9)	0.3033
	
Previous Major Health Problems	142.0(32.1)	20(25.6)	39(33.6)	52(33.1)	31(34.1)	
	
**System Factors**						

Poor Provider Experiences	374.0(88.0)	68(90.7)	96(85.7)	131(86.2)	79(91.9)	0.7793
	
Good Provider Experiences	51.0(12.0)	7(9.3)	16(14.3)	21(13.8)	7(8.1)	
	
Missing Provider Experiences	17	3	4	5	5	

### Interconceptional care participation rates

We expected that with the intensive efforts to reduce barriers, the proportion of women who attended all scheduled visits would be close to 100%. Despite having all known barriers to care addressed, only 20.6% attended all scheduled visits and 17.6% of women did not attend any of their scheduled clinic visits (Table 2). The average overall (in-clinic) participation rate for women was 52% (std 0.43) with the highest participation levels in the housing intervention (38%, std 0.43) and lowest in the infection intervention (0.06, std 0.23). (data not shown)

**Table 2:** Proportion of women by care utilization status (N = 442)

Characteristic	N (%)
No Visits	78.0(17.6)

0 to 50%	116.0(26.2)

50 to 99%	157.0(35.5)

All Visits	91.0(20.6)

### Predictors of interconceptional care participation

The Andersen Behavioral Model as a whole did not offer significant prediction of participation in interconceptional care (Wald chi sq, *p *= 0.40) under the null hypothesis that there is no relationship between the full model variables and care participation level. (Table 3, model 5)

**Table 3:** Global model building summary: PENS prediction of utilization of interconceptional care

Model specification	Number of observations	Wald Chisq	Df	*P *value*
Predisposing Only (Model 1)	352	37.6514	27	0.0835

Enabling Only (Model 2)	344	20.4951	12	0.0583

Needs Only (Model 3)	442	3.8	6	0.7037

Systems Only (Model 4)	425	2.69	3	0.4419

Full Anderson Behavior Model (Model 5)	265	49.0046	48	0.4326

Individual Models				

Insurance	442	3.1777	3	0.3650

Income	359	5.8305	3	0.1202

Social support	425	1.2121	3	0.7501

Neighborhood safety	424	3.1148	3	0.3743

Competing demands	442	0.3698	3	0.9464

Has a provider	425	2.6900	3	0.4419

Age	442	8.5929	3	0.0352

Race/ethnicity	432	1.9461	3	0.5837

Marital status	440	2.8838	3	0.4099

Education (HS grad)	442	0.1420	3	0.9864

Education (Less than HS)	442	2.8830	3	0.4100

Family size	437	0.6410	3	0.8870

Drug use	360	6.2302	3	0.1009

Alcohol use	360	1.1814	3	0.7575

Smoke	432	22.6342	3	< 0.0001

Physical health problems	442	1.8854	3	0.5965

Mental health problems	442	2.1730	3	0.5373

*This is the *p*-value associated with the specified Chi-Square statistic. Here, the null hypothesis is that there is no relationship between the any of the predictor variables and the outcome.

We examined predictors by strata of participation using the logit model. None of the model factors were sufficient to explain why women attended **none **of the scheduled interconceptional visits. (Table 4)

**Table 4:** PENS prediction of utilization of interconceptional care: odds ratio estimates (95% Wald Confidence Limits) by components and full model using logit model

Model	Utilization		
**Model 1: Predisposing Factors Only**	**No Visits**	**Some Visits**	**Most Visits**

Non-Black vs Black	1.036(0.371,2.894)	0.725(0.273,1.927)	1.248(0.526,2.959)

Age > 25 years vs Age < 25 years	1.880(0.897, 3.939)	1.964(0.985,3.914)	0.850(0.445,1.622)

Not Married vs Married	0.942(0.277, 3.202)	1.081(0.344, 3.397)	1.049(0.359,3.067)

High School education vs Greater than High School	1.594(0.662,3.837)	1.400(0.626,3.129)	1.502(0.730,3.090)

Less than High School vs Greater than High School	2.147(0.807, 5.710)	1.977(0.801,4.879)	1.430(0.628,3.255)

More than 2 family members vs 1 or 2 members	1.437(0.638,3.238)	0.676(0.330,1.385)	1.017(0.525,1.972)

Drug use vs No Drug use	1.262(0.377, 4.232)	1.530(0.531,4.411)	1.394(0.491,3.960)

Alcohol use vs No Alcohol use	0.508 (0.156,1.659)	0.479(0.171,1.344)	0.553(0.211,1.448)

Smoker vs Non-Smoker	1.300(0.526,3.210)	3.463(1.538,7.797)	1.646(0.751,3.608)

**Model 2: Enabling Factors Only**			

Income < = $20,000 vs > $20,000	1.313(0.639, 2.700)	0.931(0.488,1.776)	0.559(0.300,1.045)

No Social Support vs Has Social Support	0522(0.238,1.146)	0.822(0.423,1.597)	0.530(0.276, 1.018)

Neighborhood not safe vs Neighborhood safe	0.942 (0.457, 1.942)	1.719 (0.907, 3.261)	1.630(0.882,3.012)

Have a competing need vs no competing need	0.667 (0.325, 1.369)	0.739(0.387, 1.411)	0.783 (0.421, 1.455)

**Model 3: Needs Factors Only**			

Poor Physical Health vs. Good Physical Health	1.014(0.340,3.018)	1.558 (0.612, 3.966)	1.549(0.622,3.857)

Poor Mental Health vs. Good Physical Health	1.052(0.472,2.345)	1.144 (0.557,2.349)	0.744(0.366, 1.513)

**Model 4 Systems Factors Only**			

No Healthcare Provider vs Has a Healthcare Provider	0.862 (0.288, 2.579)	0.532 (0.209, 1.358)	0.553 (0.225, 1.360)

**Full Model**			

Non-Black vs Black	0.481(0.133,1.748)	0.482(0.153,1.512)	0.922(0.332,2.557)

Age > 25 years vs Age < 25 years	1.630(0.675,3.932)	1.747(0.763,3.997)	0.798(0.367,1.738)

Not Married vs Married	0.983(0.261,3.710)	1.141(0.318,4.092)	1.156(0.341,3.920)

High School education vs Greater than High School	1.389(0.489,3.949)	1.263(0.488,3.267)	1.315(0.547,3.160)

Less than High School vs Greater than High School	2.194(0.662,7.276)	1.600(0.517, 4.951)	1.028(0.355,2.978)

More than 2 family members vs 1 or 2 members	1.518(0.593, 3.887)	0.789(0.338,1.845)	1.109(0.504,2.438)

Drug use vs No Drug use	0.990(0.211, 4.633)	2.265(0.606, 8.463)	1.527(0.408, 5.713)

Alcohol use vs No Alcohol use	0.258(0.058,1.139)	0.321(0.091,1.126)	0.386(0.114,1.308)

Smoker vs Non-Smoker	1.965(0.615,6.276)	5.050(1.741,14.650)	2.109(0.739,6.023)

Income < = $20,000 vs > $20,000	1.247(0.516, 3.012)	1.165(0.502,2.702)	0.634(0.291,1.381)

No Social Support vs Has Social Support	0.724(0.285,1.842)	0.602(0.251,1.444)	0.667(0.300, 1.482)

Neighborhood not safe vs Neighborhood safe	1.103(0.468,2.598)	1.838(0.828,4.082)	1.984(0.941,4.179)

Have a competing need vs no competing need	0.650(0.273,1.547)	0.976(0.429,2.221)	0.809(0.378,1.732)

Poor Physical Health vs. Good Physical Health	0.867(0.192, 3.921)	1.229(0.306,4.936)	1.156(0.309,4.332)

Poor Mental Health vs. Good Mental Health	1.168(0.369,3.692)	0.683(0.229,2.035)	0.541(0.187,1.562)

No Healthcare Provider vs Has a Healthcare Provider	0.847(0.229,3.136)	0.592(0.183,1.916)	0.739(0.239,2.282)

Logistic regression model assuming a multinomial distribution

Among women who attended "some visits" (1-50% of visits), *smoking *was a significant predictor, where smokers were 3.4 times more likely to attend some vs. all visits (e.g. smokers are more likely to miss some scheduled visits).

Among those who attended most visits (> 50% of visits), there were no clear predictors of participation in care. "Neighborhood perceived as unsafe" was marginally predictive, where the women who perceived an unsafe neighborhood were less likely to attend all visits. (Table 4)

### Predictive power of model components

#### PENS

We tested the model components: predisposing, enabling, needs and systems factors separately to determine if any component by itself could predict interconceptional care participation. Only Enabling factors as a group (income, social support, neighborhood safety and competing needs) offered marginally significant predictive power (*p *= 0.058). (Table 4)

### Predictive power of individual model variables

When we examined individual components of the full model, age (*p *= 0.035) and smoking use (*p *= < 0.001) significantly predicted participation in interconceptional care visits (Table 3). We looked at smokers as a separate strata (data not shown) and found several differences with non-smokers. Smokers were less educated, had larger family sizes, were more likely to use drugs or alcohol, had higher income yet lower social support than non- smokers, and were more likely to report poor mental health or prior major health problems.

## Discussion

### Summary of key findings

The Andersen Behavioral Model has been used previously to identify barriers to care participation and contains a comprehensive set of factors known to influence utilization of care [18]. In this study, we applied the model to a population that differed from previous studies in two fundamental ways: (1) the population of women was receiving *preventive care in the interconceptional period*, and (2) as part of the care protocol, *all traditionally known barriers to care were addressed *to facilitate participation in the clinical trial. We expected to be able to identify which factors above and beyond those already addressed as part of the care protocol exerted impact on women's participation in interconceptional care.

While no factors clearly distinguished the women who missed every one of their scheduled visits compared to those who attended all visits, smoking acted as a barrier, significantly increasing the likelihood those women would miss over half of scheduled visits (compared to ALL visits).

### Strengths and limitations

The disadvantage of self-reporting lies in the possibility of reporting bias. We expect some underreporting of substance use and possibly in self -report of quality of prior experiences with health care providers, but there is no reason to suspect that the underreporting varies by level of care participation.

All women were not assigned to the same constellation of interventions and some were scheduled for more invasive appointments than others. Burden of intervention may be a factor in women's ability to attend. That is, the more appointments they are scheduled for, or the more invasive the intervention, the more likely they may be to miss some appointments. We did not consider the type of intervention, only the aggregate participation, and looked only at the number of visits attended as a proportion of the number scheduled. We assessed the correlation between number of scheduled visits and participation rates (data not shown) and found that there is not a linear relationship between the two. For example, the mean number of scheduled visits among those with a 0 participation rate was 2.26, while the mean was 8.94 scheduled visits for those who attended "some" visits, 9.77 for those who attended "most" visits, and 2.58 for those who attended "all" visits. Also, the number of scheduled visits is not a direct marker for intervention intensity because a subject may have had a second visit scheduled because she missed the previous appointment. As such, a high number of scheduled visits could represent several re-scheduled appointments. Our categorization of the outcome variable may also be a limitation. These groupings were used to delineate participation patterns that have some practical conceptual meaning (none, some, most, all). Fewer categories may have yielded a more parsimonious model, but would not improve conceptual interpretation. For example, aggregating the "Some" and "Most" groups is not conceptually valid because we would be looking at a difference between those who attend 1% and those who attend 99% of visits. We tested an aggregation of the "Most" and "All" categories to assess for differences. In this case, the mean visits and standard errors differ significantly between these categories so information would likely be lost by this aggregation. We did not find any significant changes in the findings reported. Finally, an inherent problem with the Andersen Behavioral Model is that it only predicts *utilization *of organized health care, which assumes that the intervention effectiveness is only based on what happens in the clinical setting. For many of the indicated interventions, follow-up activities are required in the home and the community, thus utilization of organized care may not fully predict impacts of the care received. In these analyses, we are not looking specifically at outcomes of care, only at utilization of organized, clinic-based interconceptional care.

The Andersen Behavioral Model is a holistic theoretic model and incorporates multiple levels of contextual and behavioral factors to explain health care utilization. In addition, this study took advantage of the unique opportunities presented by the parent study which applied a comprehensive set of evidence-based risk reduction approaches during *the interconceptional period*. This is the first study to measure predictors of participation in a large-scale, organized, comprehensive interconceptional care intervention among a mostly African American, urban and vulnerable population. In addition, an evaluation of participation under the best-case scenario (that is--where all known barriers to participation have been addressed) is also undertaken.

### Interpretation

The parent clinical trial recruitment demonstrated that addressing barriers to health service access might generate high participation of mostly African American women in interconceptional care [15]. However, when it comes to the long-term participation required for most forms of preventive care, we find that other unknown factors continue to affect women's willingness and ability to participate consistently in these services.

Barriers to types of preventive care such as ICC may differ considerably from the barriers to prenatal care. Pregnancy provides a motivational force and immediacy of concern for the baby's health to spur participation. Additionally, social norms exist which create a negative view of women who do not participate in PNC. No equivalent motivators or deterrents exist for interconceptional care and we have no studies that have identified barriers specific to this type of women's preventive care. For this study, we tested *a priori *assumptions that the same barriers would apply to both PNC and ICC participation. Studies delineating the barriers to PNC have evolved over time. Models of PNC participation started out including a conglomeration of individual factors describing populations at risk (young, less educated, single, large family size) [19,20]. These models were later modified to include behavioral factors (substance use, stress, low social support) [20-22]. Later, ecologic models were used, including factors which exist outside of the personal domain as predictors of utilization. These include contextual factors that influence women's ability to *get away from competing demands *(job demands, childcare needs) [22-24], *to get to the health care site *(transportation, income) [19,21,23], and factors influencing the *quality of treatment once in the health care *site (provider availability, wait times, hours of operation, discrimination) [23,24]. The Andersen Behavioral models may be the first to incorporate these multiple levels including barriers that exist outside of individual control [18]. Inclusion of systemic and contextual factors as barriers or facilitators of the receipt of care is significant because it can potentially change the locus of intervention from the individual to systems. While theoretically, the domains of the Andersen model capture an appropriate and broad conceptualization of factors associated with care utilization, it is **how **the components are operationalized that ultimately define how well it will predict preventive care utilization. The specific factors included in the model must reflect the unique contexts and experiences of the population under study [25,26]. While we operationalized all suggested model variables, and other known barriers were addressed as part of the care protocol, none of the currently known factors accounted for the non-participation of some women receiving free care nor do they explain the full participation (100%) of other women. We conclude that although we had the data to operationalize almost all variables suggested as relevant to vulnerable populations (we lacked only information on language barriers and homelessness length), these models still do not capture all of the barriers and facilitators specific to utilization of interconceptional care utilization for this population of urban, mostly African American women. These factors can only be identified through a deep contextual look at the daily lives of the women.

## Conclusions

Advocacy is currently underway to develop and promote a system for delivery of ICC [11,12,27]. Development of a knowledge base of the barriers to interconceptional preventive care is critical and timely to inform the process of building this preventive service. Careful attention must be paid to ensuring that barriers do not impede *any *vulnerable population group's access and participation. Health promotion strategies must not only increase demand for ICC service, but must also proactively identify individual, contextual and structural barriers, then develop structures and processes for delivery of care that facilitate equitable access for all women. Individual behavioral strategies tend to be divorced from the contextual or structural mediators of health care utilization. Marketing, for example, is an individual-focused behavioral strategy that may disproportionately benefit higher income and higher educated women because these women are less likely to face social or economic barriers to participation once they are informed of the need and availability of the service. We showed that other factors, above and beyond traditional barriers to care, possibly contextual or structural factors, continue to impact on participation in ICC care for urban women. The challenge remains for new paradigms to be developed to capture the full range of barriers affecting interconceptional preventive health care participation, particularly for vulnerable population groups. Once these factors are identified, inter- and preconceptional care can be structured and delivered to ensure equitable access and will only then hold promise to reduce disparities in preterm-related mortality.

## Abbreviations

ABM: Andersen behavioral model; BMI: Body mass index; FIM: Feto-infant mortality; ICC: Interconceptional care; IM: Infant mortality; IRB: Institutional review board; LB: Live biirth; MH/P: MHP: Maternal Health/Prematurity; PCC: Preconception care; PENS: Predisposing conditions, Enabling factors, Need factors, Systemic factors; PNC: Prenatal care; POM: Proportional odds model; PPOR: Perinatal periods of risk; PTB: preterm birth.

## Competing interests

The authors have no financial or non-financial competing interests.

## Authors' contributions

VH conceived and designed the study, participated in analysis and interpretation. JC and DW made substantial contributions to the data acquisition, interpretation, manuscript preparation, AA and AA made substantial contributions toward analysis and interpretation and manuscript preparation, DR made substantial contributions toward interpretation and manuscript preparation. All authors read and approved the final manuscript.

## Funding sources

Research funded by: National Institutes of Health (K01HD54440) and Pennsylvania State Department of Health, Grant # ME410002073.

These funders played no role in decisions regarding the analysis, interpretation or manuscript preparation.

IRB approved by UNC-CH IRB project # 07-0857.

## Pre-publication history

The pre-publication history for this paper can be accessed here:

http://www.biomedcentral.com/1471-2458/12/93/prepub
